# Association between neutrophil to lymphocyte ratio and the risk of vertebral fracture in patients with osteoporosis: a systematic review and meta-analysis

**DOI:** 10.3389/fendo.2026.1739898

**Published:** 2026-02-18

**Authors:** Dezhi Xu, Jiaxin Wang

**Affiliations:** 1 Department of Spine Surgery, Yongkang Orthopedic Hospital, Yongkang, Jinhua, Zhejiang, China; 2 Department of Pain Rehabilitation, Yongkang Orthopedic Hospital, Yongkang, Jinhua, Zhejiang, China

**Keywords:** inflammatory biomarkers, meta-analysis, NLR, osteoporosis, osteoporotic fractures, risk stratification, systematic review, vertebral fracture

## Abstract

**Background:**

Vertebral fractures, which account for 40% of osteoporotic fractures, often lack early clinical symptoms. Previous studies have shown that neutrophil-to-lymphocyte ratio (NLR) has potential predictive value for vertebral fractures, but evidence-based conclusions are lacking. This meta-analysis is the latest to evaluate the link between NLR and vertebral fracture risk in patients with osteoporosis.

**Methods:**

We systematically searched PubMed, Embase, Web of Science, Cochrane, Wanfang and CNKI (up to March 2025). The search was performed using the following keywords: “Neutrophils”, “Lymphocytes”, “Osteoporosis”, and “Fracture”. Odds ratio (OR) and standardized mean difference (SMD) with 95% confidence intervals (CIs) were used for the data synthesis of categorical and continuous variables, respectively. Sensitivity analysis was performed to explore the stability of the results and possible sources of heterogeneity. All analyses were performed using Review Manger 5.4 and STATA 15.0.

**Results:**

Six observational studies (n=938) were included. Categorical data showed a significantly higher vertebral fracture risk in high-NLR groups (OR = 3.75, 95% CI:1.79-7.86; *P* = 0.0005). However, continuous data revealed no significant NLR difference between fracture and non-fracture groups (SMD = 0.51, 95% CI: -0.06-1.08; *P* = 0.08). Sensitivity analysis revealed that when the data of Li et al., 2023 were excluded, the results of continuous data shifted from insignificant to significant (SMD: 0.27; 95% CI: 0.08, 0.46; *P* = 0.004), and the heterogeneity decreased significantly.

**Conclusion:**

Higher NLR values ​​in osteoporotic patients are significantly associated with an increased risk of vertebral fractures, but the strength of this evidence is limited by high heterogeneity and the instability of results from continuous variables. Current findings support NLR as a potential inflammation-related biomarker for vertebral fractures, but its clinical application requires careful interpretation. Future research should focus on conducting more high-quality, large-sample prospective studies to standardize NLR thresholds and validate its practical value in risk stratification for osteoporotic fractures.

**Systematic Review Registration:**

https://www.crd.york.ac.uk/PROSPERO/view/, identifier CRD420251023391.

## 1 Introduction

Osteoporosis, a systemic skeletal disorder marked by osteopenia and degradation of bone microarchitecture, has emerged as a major global public health concern (1–3). The International Osteoporosis Foundation (IOF) reports that osteoporotic fractures occur every 3 seconds worldwide, with vertebral fractures representing approximately 40% of these fractures. Individuals who have experienced vertebral fractures face a nearly fivefold increased risk of subsequent fractures compared to those with non-vertebral fractures (4). Epidemiological data from China show that the prevalence of vertebral fractures in individuals over 50 years of age is approximately 15%, but only 20%-30% of those with vertebral fractures exhibit typical clinical symptoms, leading to a misdiagnosis rate as high as 60% (5, 6). Epidemiological studies in Europe show that the prevalence of vertebral fractures in people over 50 years of age ranges from 12% to 18%, increasing significantly with age (7, 8). In the United States, data from the National Health and Nutrition Examination Survey (NHANES) indicates that the prevalence of vertebral fractures is approximately 25% in women aged 50 and older, and approximately 10% in men, highlighting the widespread disease burden (9, 10). Although the FRAX tool is commonly used in clinical settings to assess osteoporotic fracture risk, its sensitivity for predicting vertebral fractures is limited, particularly in patients with early-stage osteoporosis who lack additional risk factors (11). This underscores the need for the identification of new biomarkers to enhance the current fracture risk assessment framework.

While the FRAX tool is widely used globally, other validated fracture risk assessment tools, such as QFracture and the Garvan calculator, each have their own characteristics. QFracture, developed in the UK, has the advantage of incorporating a broader range of clinical risk factors and assessing risk without relying on bone mineral density data (12). However, its model is primarily based on UK population data, and its applicability and calibration in other countries may require further validation. The Australian Garvan calculator specifically emphasizes and quantifies the significant impact of fall history and previous fracture counts on fracture risk, providing 5-year and 10-year fracture risk predictions (13). However, it covers relatively fewer clinical risk factors, and its ability to differentiate in older populations may be limited. These tools share several challenges, often struggling to capture and integrate dynamic biological information reflecting a patient’s real-time pathophysiological state. Therefore, exploring novel biomarkers holds promise for providing valuable and readily available supplementary information to existing, relatively static clinical risk assessment systems, potentially enabling more sensitive identification of patients at extremely high fracture risk.

Although osteoporosis can lead to various fracture types, this study focuses specifically on vertebral fractures, primarily due to their unique clinical importance: vertebral fractures are the most common type of osteoporotic fracture, accounting for approximately 40%, and because they often lack typical clinical symptoms in the early stages, the rate of underdiagnosis and misdiagnosis is as high as 60%, thus delaying treatment (14); more importantly, patients with vertebral fractures have a nearly 5-fold increased risk of subsequent fractures, significantly increasing disability and mortality rates, placing a heavy burden on patients’ quality of life and the healthcare system (15, 16). Therefore, focusing on vertebral fractures helps to reveal the predictive value of inflammatory biomarkers in this high-risk but easily overlooked outcome, providing targeted evidence for early intervention.

In recent years, the role of chronic low-grade inflammation in the pathological process of osteoporosis has gained growing interest. As a marker of systemic inflammation, the neutrophil to lymphocyte ratio (NLR) has been shown to be closely linked to an imbalance in bone metabolism (17–19). Research shows that neutrophils activate the RANKL/osteoporosis G signaling axis by secreting IL-17, thereby enhancing osteoclast differentiation (20). Meanwhile, matrix metalloproteinase 9 (MMP-9) released by neutrophils can directly degrade type I collagen, leading to a reduction in trabecular connectivity (21). Lymphocyte subsets, such as regulatory T cells, counteract osteoclast activity by secreting IL-4 and IL-10, thus preserving bone remodeling homeostasis (22). Clinical cohort studies have revealed that peripheral blood NLR in osteoporosis patients is significantly elevated compared to healthy controls, and negatively correlates with femoral neck bone density (23–27). Collectively, these findings suggest that NLR acts as a promising biomarker for predicting vertebral fractures by reflecting the inflammatory status of the bone microenvironment.

The clinical value of the NLR in fracture risk prediction has been established across various disciplines. For instance, in cardiovascular diseases, elevated NLR has been strongly linked to an elevated risk of major adverse cardiovascular events (28). However, its predictive accuracy for osteoporosis-related vertebral fractures remains contentious: a prospective cohort study involving 92 patients with post-menopausal osteoporosis found no significant correlation between NLR and the risk of vertebral fractures (29), while another prospective cohort study (n=80) identified NLR as an independent risk factor for osteoporotic vertebral fractures (OR = 13.229, 95%CI 4.167-41.998) (30). This heterogeneity may be due to differences in study design, including the timing of NLR detection (acute inflammatory phase vs stable phase), fracture diagnostic criteria (radiological diagnosis vs clinical symptoms), and control of confounding factors (such as history of glucocorticoid use). Therefore, evidence-based research is needed to integrate global observational studies, not only to quantitatively assess the overall association strength between NLR and the risk of vertebral fractures in osteoporotic patients, but also to explore the sources of discrepancies in existing research findings through heterogeneity analysis. Clearly identifying and assessing these heterogeneities is of great value in promoting the clinical translation and application of NLR as a predictive biomarker for vertebral fractures.

This latest study aims to integrate global observational research evidence and quantitatively evaluate the relation between NLR and the risk of vertebral fractures in osteoporotic patients. The results will provide important evidence for improving the risk stratification of osteoporotic fractures and formulating individualized intervention strategies, while opening up new perspectives for the translational application of inflammatory mechanisms in bone metabolic diseases.

## 2 Methods

### 2.1 Literature search

This meta-analysis was conducted following the PRISMA 2020 guidelines (31) and was pre-registered in PROSPERO (CRD420251023391). To identify relevant studies assessing the association between NLR and vertebral fracture risk in osteoporosis patients, we performed a systematic search across PubMed, Embase, Web of Science, Cochrane, Wanfang, and CNKI up to March 2025. The search was performed using the following keywords: “Neutrophils”, “Lymphocytes”, “Osteoporosis”, and “Fracture”. A detailed search strategy for PubMed is provided below: (((((“Neutrophils”[Mesh]) OR ((((Neutrophil) OR (Polymorphonuclear Leukocyte)) OR (LE Cell)) OR (Neutrophil Band Cell))) AND ((“Lymphocytes”[Mesh]) OR (((Lymphocyte) OR (Lymphoid Cells)) OR (Lymphoid Cell)))) AND (Ratio)) AND ((“Osteoporosis”[Mesh]) OR (Osteoporoses))) AND ((“Fractures, Bone”[Mesh]) OR (((((Bone Fracture) OR (Broken Bones)) OR (Broken Bone)) OR (Fracture)) OR (Fractures))). In addition, we thoroughly reviewed the reference lists of all included studies to identify any possibly relevant articles. Two authors independently retrieved and assessed the studies for eligibility, with any differences in the selection process being resolved through discussion. An outline of the literature search strategy is available in **Supplementary Table S1**.

### 2.2 Inclusion and exclusion criteria

To reduce bias, longitudinal design studies were included in this meta-analysis. Studies were considered eligible when fulfilled the following criteria: (1) the study design was a cohort study, a case-control study, or a randomized controlled trial; (2) participants were diagnosed with osteoporosis; (3) the study investigated the relationship between NLR and vertebral fracture risk; (4) at least one outcome measure (categorical or continuous) was reported; and (5) sufficient data were available to conduct a multivariate analysis of the odds ratio (OR) or standardized mean difference (SMD) with a 95% confidence interval (CI).

We excluded non-original articles (such as letters, replies, comments, corrections, and abstracts), review articles, unpublished research, study protocols, and studies lacking sufficient data.

### 2.3 Data abstraction

Two authors independently performed data extraction, with any conflicts being addressed by a third author. The following information was collected from the studies: first author’s name, published year, country of study, study design, study population, sample size, gender, age, multivariate analysis OR and mean with standard deviation (SD), NLR cut-off value. Record the completeness of research report data. In cases of missing key data, our strategy is to first contact the corresponding author of the original study via email to retrieve the unreported data. If contact with the author is unsuccessful or the author is unable to provide the data, we assess the impact of the missing data on the pooled analysis. If the missing data is key data that cannot be obtained through simple extrapolation, such as the variance of the effect size or confidence intervals, and if the absence could introduce significant bias into the pooled analysis, we plan to exclude the study in the sensitivity analysis to assess its impact on the stability of the overall results.

### 2.4 Quality evaluation

To assess methodological quality, the Newcastle-Ottawa Scale (NOS) was employed. Only studies achieving a score of 6 or higher (out of 9), indicating high quality, were eligible for inclusion in the quantitative synthesis. Studies scoring below this threshold were excluded from the meta-analysis. Two reviewers independently performed the quality assessment, and any disagreements were settled through consensus-based discussion.

### 2.5 Statistical analysis

Review Manager software (version 5.4.1) was employed to perform the meta-analysis. For data synthesis, OR with 95% CIs were applied to categorical variables (OR is suitable for categorical variables, directly quantifying the relative magnitude of vertebral fracture risk and facilitating clinical interpretation), while SMD with 95% CIs were employed for continuous variables (SMD is suitable for continuous variables, standardizing the measurement scales of different studies, eliminating unit differences, and achieving effective pooling). To assess heterogeneity across outcomes, Cochran’s Q test (χ²) and the I² statistic were utilized. A χ² P-value below 0.1 or an I² value exceeding 50% indicated substantial heterogeneity. The random-effects model was adopted to calculate the pooled ORs and SMDs. In addition, sensitivity analysis assesses the impact of individual studies on the overall results by recalculating the pooled effect size after removing each study one by one, thereby verifying the robustness of the conclusions. Potential publication bias was further explored using funnel plots generated in Review Manager (version 5.4.1), and Egger’s regression test was performed by employing Stata software (version 15.1, StataCorp, College Station, TX, USA) for outcomes comprising three or more studies. A P-value less than 0.05 was considered as an indicator of a significant publication bias.

## 3 Results

### 3.1 Literature retrieval and study characteristics

**Figure 1:**
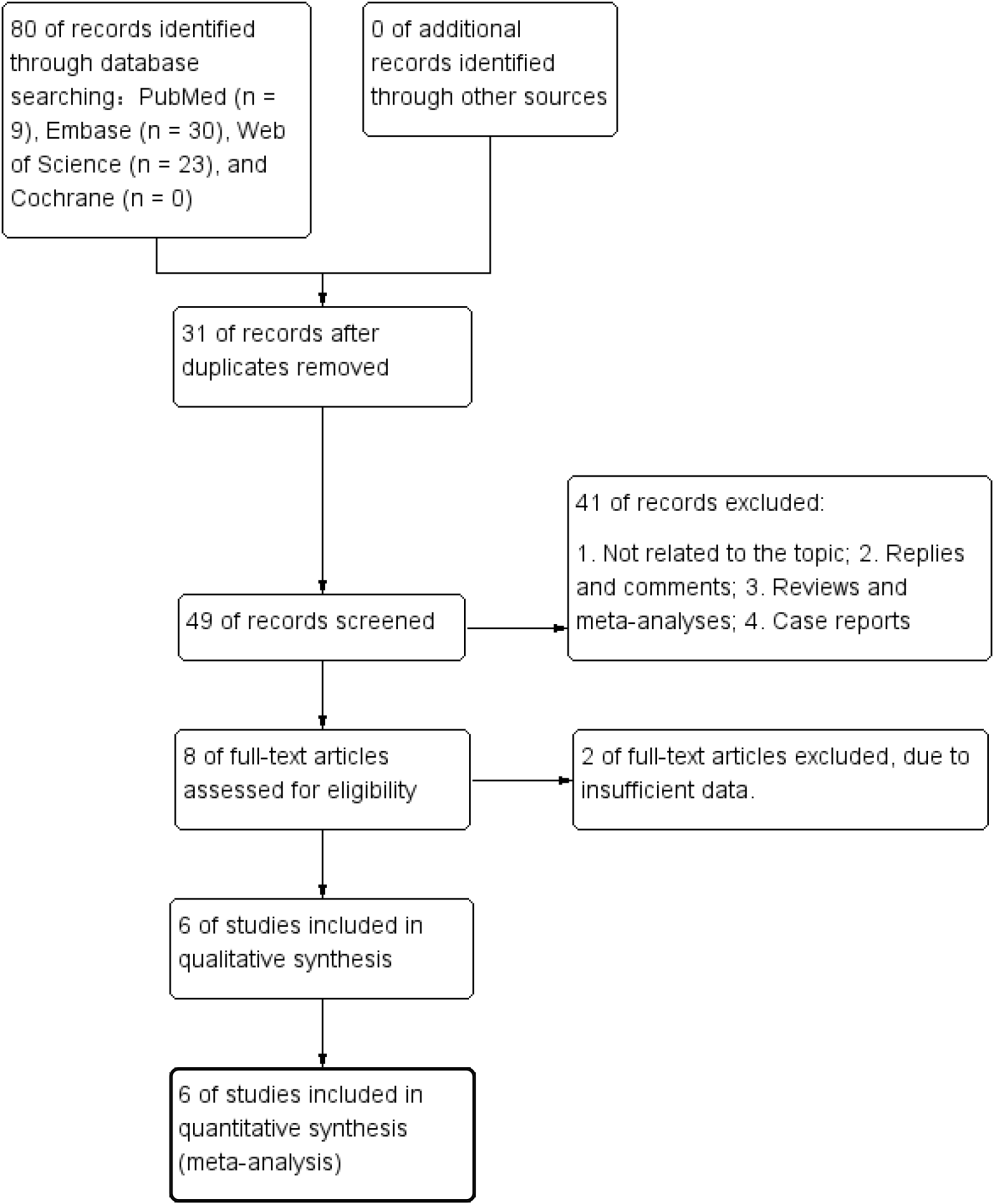
This study systematically demonstrates the complete workflow for identifying relevant literature through both computer and manual searches, and ultimately determining the eligible studies for inclusion in meta-analysis. The workflow follows the PRISMA guidelines, detailing the number of records initially retrieved from each database, the number of records after removing duplicates, the number of documents retained after title and abstract screening, the number of documents excluded during the full-text evaluation stage and the specific reasons for exclusion, and the final number of studies included in both qualitative and quantitative meta-analyses. This figure ensures the transparency and reproducibility of the literature screening process.

**Figure 1** presents a flowchart outlining the process of literature identification and selection. Through a systematic search, 80 relevant studies were identified across various databases, including PubMed (n = 9), Embase (n = 30), Web of Science (n = 22), Cochrane (n = 0), Wanfang (n = 10), and CNKI (n = 9). Following the removal of duplicate entries, 49 titles and abstracts underwent screening. In the end, six cohort studies encompassing a total of 938 patients fulfilled the criteria of eligibility and were included in the meta-analysis (29, 30, 32–35). **Table 1** provides a summary of the characteristics and quality evaluations of the selected studies.

**Table 1:** Characteristics and quality evaluation of included studies.

Study	Country	Study design	Population	No. of patients	Gender		Mean/median age	NLR cut-off	NOS score
					Male	Female			
Cal 2024	Turkey	Retrospective	Patients aged 50 years or older who presented with osteoporotic vertebral fractures and underwent kyphoplasty	50	11	39	68.8	NA	7
Fang 2020	China	Prospective	Patients with postmenopausal osteoporosis	92	0	92	60.5	3.64	7
Fu 2024	China	Retrospective	Patients diagnosed with osteoporosis	310	37	273	67.38	2.307	8
Gou 2024	China	Retrospective	Patients diagnosed with osteoporosis	176	NA	NA	68	NA	8
Li 2023	China	Retrospective	Patients with postmenopausal osteoporosis	230	0	230	62.15	NA	8
Zhu 2022	China	Prospective	Patients diagnosed with osteoporosis	80	32	48	NA	NA	8

### 3.2 NLR and the risk of vertebral fracture (categorical variables)

**Figure 2:**
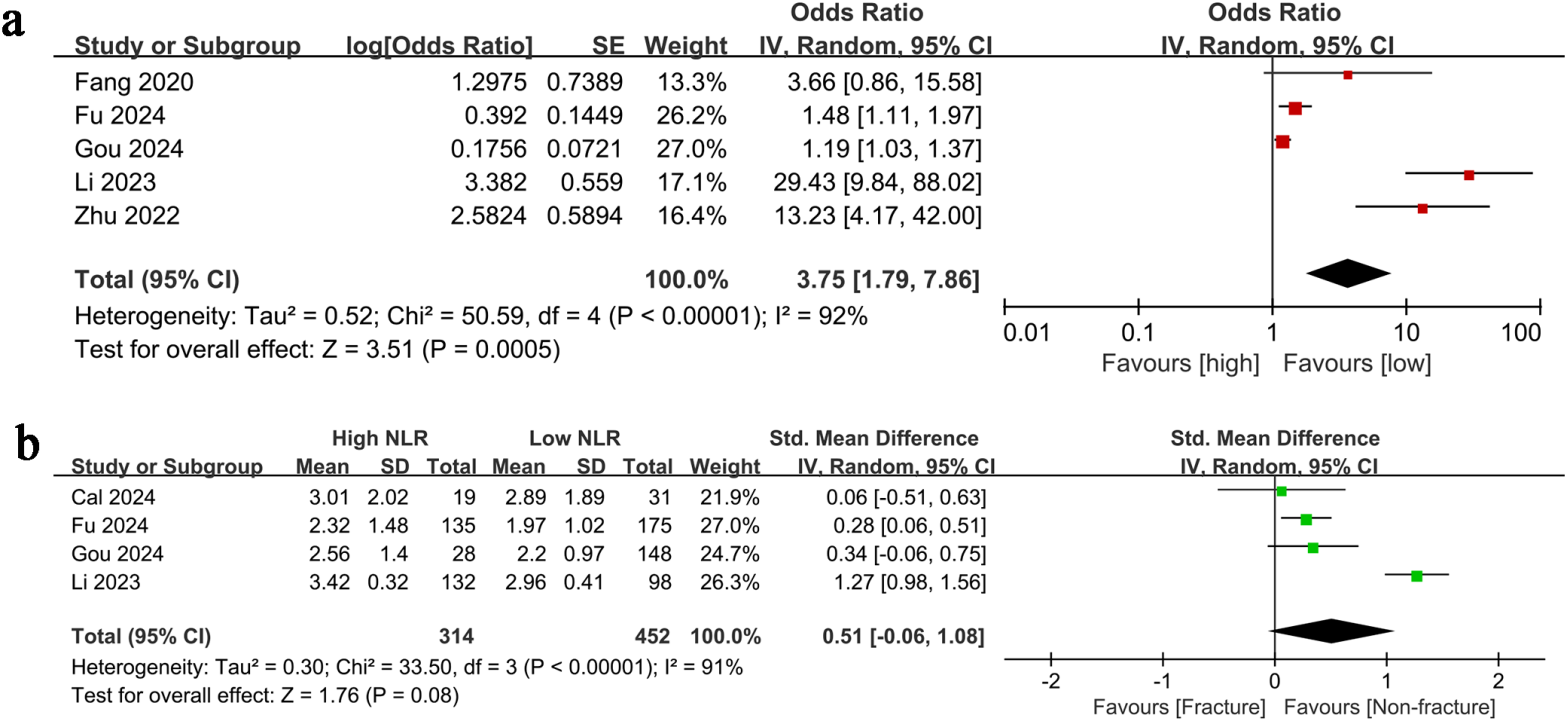
Forest plots of the results of **(A)** categorical (this forest plot presents the results of a pooled analysis of the relationship between NLR and vertebral fracture risk when NLR is used as a categorical variable (high vs. low) based on a random-effects model. The plot lists the name of each included study, the effect size and its weight as odds ratio (OR) and 95% confidence interval (CI), and the final pooled effect size (indicated by a diamond symbol). The vertical dashed line in the plot marks the null line (OR = 1). A pooled OR > 1 and a 95% CI not containing 1 indicates a significant association between high NLR and increased risk of vertebral fracture. The plot captions also include the I² statistic for assessing heterogeneity among studies) and **(B)** continuous variables [This forest plot presents the results of a pooled analysis of the relationship between NLR and vertebral fracture risk when NLR is treated as a continuous variable. Data were pooled using a random-effects model, with effect sizes expressed as standardized mean difference (SMD) and its 95% confidence interval (CI). The plot includes the SMD, 95% CI, and weights for each included study, as well as the final pooled SMD. The vertical dashed line represents the null line (SMD = 0). A pooled SMD > 0 suggests that NLR levels may be higher in fracture patients than in non-fracture patients, but a 95% CI of 0 indicates no statistical significance. The plot also reports the heterogeneity assessment results (I² value)].

Results of categorical variables were synthesized from 5 studies, and meta-analysis of multivariate data demonstrated a notably higher risk of vertebral fracture in the group with high NLR in comparison with the group with low NLR (OR: 3.75; 95% CI: 1.79, 7.86; *P* = 0.0005). A significant heterogeneity was observed (*I*^2^ = 92%, *P* <0.00001) (**Figure 2A**).

### 3.3 NLR and the risk of vertebral fracture (continuous variables)

Results of continuous variables were synthesized from 4 studies, and meta-analysis revealed a similar NLR level in the fracture and non-fracture groups (SMD: 0.51; 95% CI: -0.06, 1.08; *P* = 0.08). A significant degree of heterogeneity was identified (*I*^2^ = 91%, *P* <0.00001) (**Figure 2B**).

### 3.4 Publication bias and sensitivity analysis

**Figure 3:**
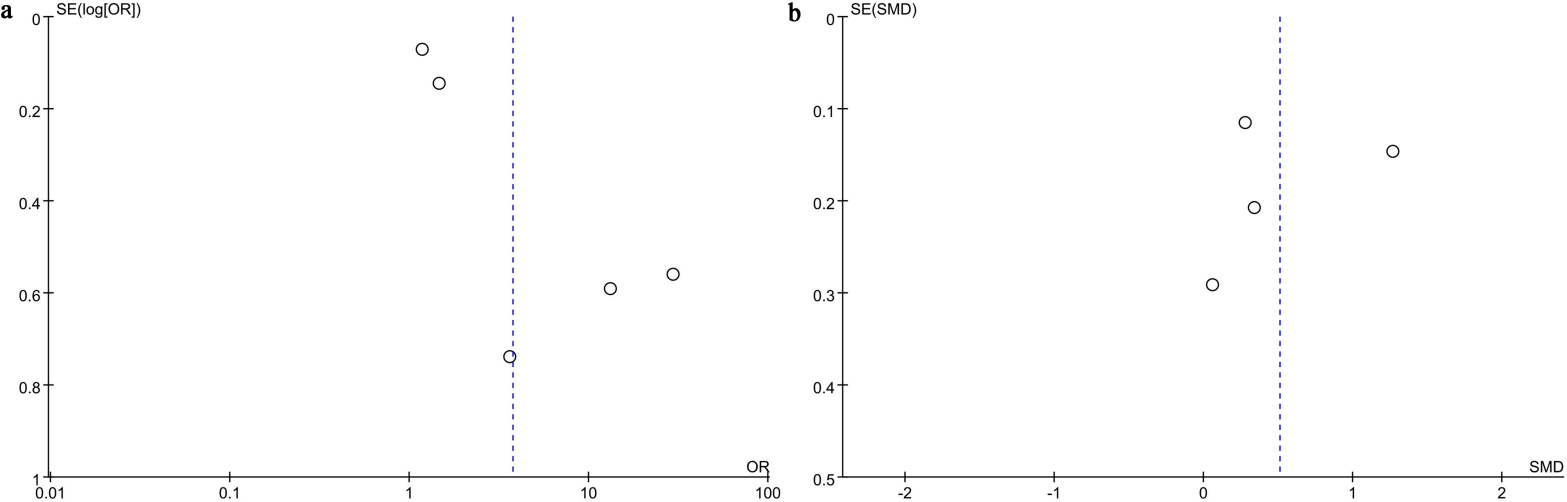
Funnel plots of **(A)** categorical and **(B)** continuous variables. This funnel plot visually assesses the presence of publication bias in relevant studies. The scatter points in the plot represent the relationship between the effect size of each included study and its standard error. Ideally, in the absence of publication bias, the scatter points should exhibit an inverted, symmetrical funnel-shaped distribution. Significant asymmetry in the graph suggests the potential presence of unpublished studies with negative results.

**Figure 4:**
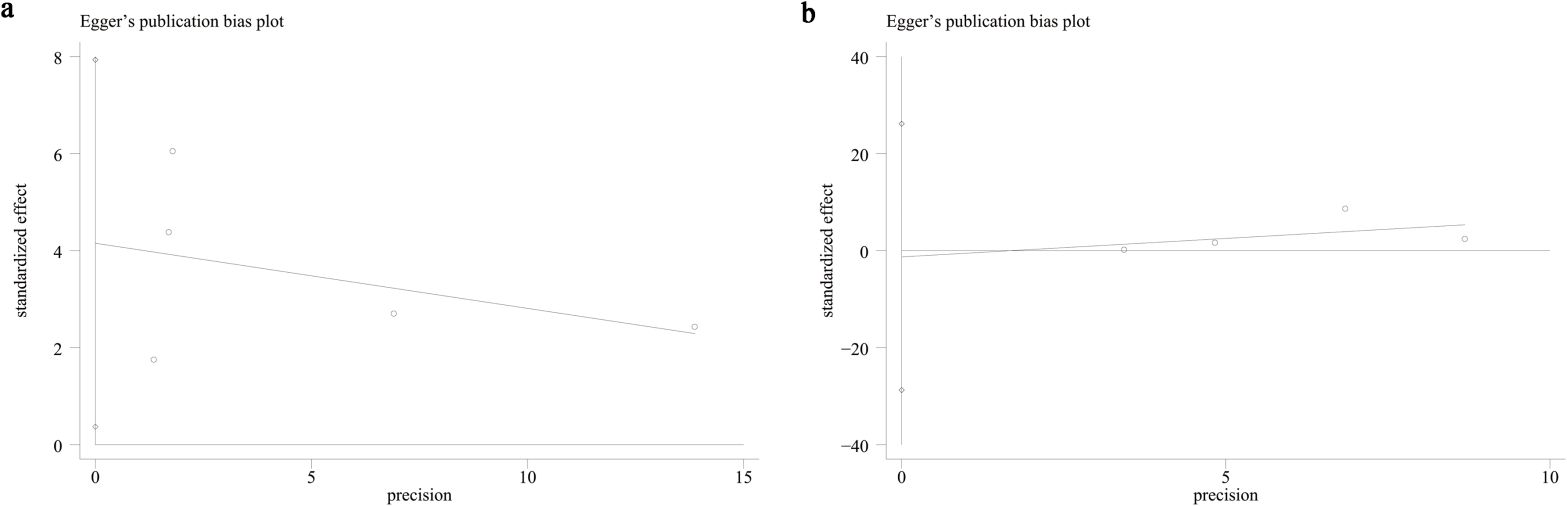
Egger’s test plot **(A)** categorical and **(B)** continuous variables.

**Figure 5:**
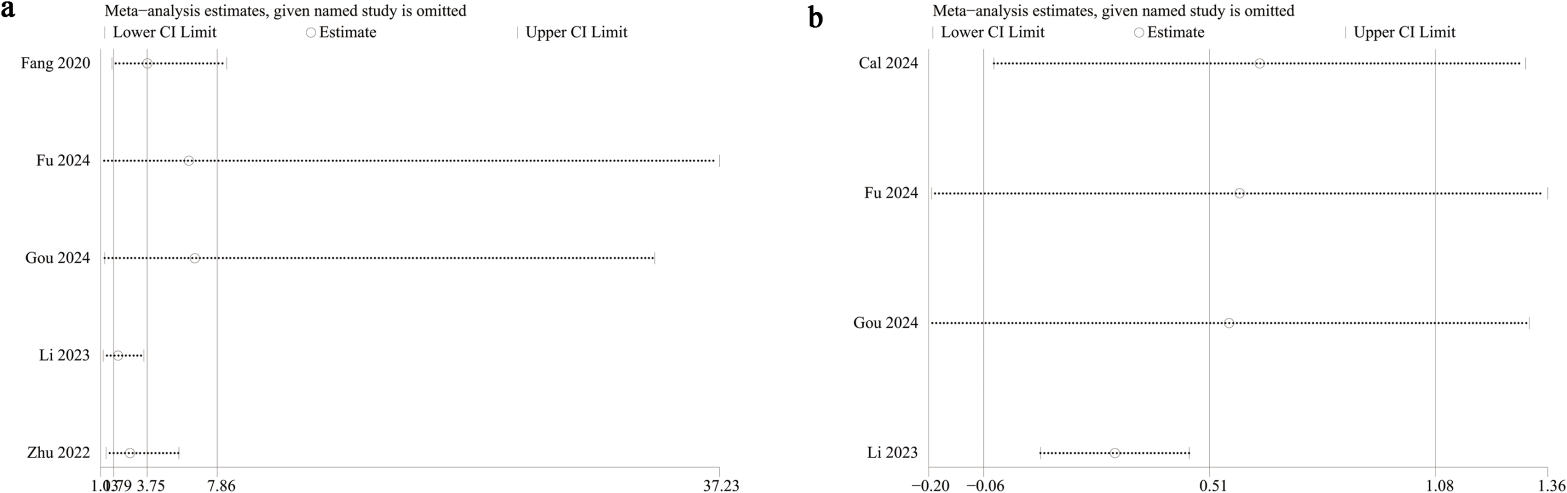
Sensitivity analysis of the **(A)** categorical and **(B)** continuous variables. This figure tests the robustness of the meta-analysis results by recalculating the pooled effect size after removing each included study one by one. The figure shows the change in the pooled effect size after removing a particular study. If the point estimate and confidence interval of the pooled effect size do not change directionally or significantly after removing any individual study, the original results are considered robust. Conversely, it suggests that the results may be overly sensitive to a specific study. This analysis helps assess the degree of influence of individual studies on the overall conclusions.

Funnel plots and Egger’s regression tests were employed to evaluate possible publication bias across both categorical and continuous variables. The funnel plot (**Figure 3A**) and Egger’s test (P = 0.04, **Figure 4A**) identified significant publication bias in categorical variables. In contrast, no evidence of bias was detected for continuous variables, as neither the funnel plot (**Figure 3B**) nor Egger’s test (P = 0.859, **Figure 4B**) suggested any risk of bias. Additionally, sensitivity analyses were performed by sequentially excluding individual cohort studies to evaluate their influence on the pooled OR and SMD. The results showed that for categorical variables, the overall OR remained stable after the sequential exclusion of each study (**Figure 5A**). However, when the data of Li 2023 (35) were excluded, the results of the continuous variables shifted from insignificant to significant (SMD: 0.27; 95% CI: 0.08, 0.46; *P* = 0.004), while heterogeneity dropped from 91% to 0% (**Figure 5B**), indicating that this outcome was significantly unstable.

## 4 Discussion

This study found through a meta-analysis of 6 studies that osteoporotic individuals with high NLR had a notably higher risk of vertebral fractures than those with low NLR, and sensitivity analysis confirmed the stability of this result. However, the continuous variable obtained a negative result, indicating that patients with vertebral fractures and non-vertebral fractures had similar NLR levels. However, it is important to note that the sensitivity analysis of continuous variables revealed that this result was not stable. When the data of Li et al., 2023 were excluded, the results shifted from insignificant to significant, and the heterogeneity decreased significantly. The results also indicated that the NLR levels in individuals with vertebral fractures were significantly higher compared to those with non-vertebral fractures, supporting the results of categorical variables. Therefore, based on the existing evidence, it can be inferred that NLR is indeed an effective inflammatory marker for vertebral fractures in osteoporotic patients, a finding that is consistent with most previous studies (30, 33–35).

The inconsistency between the categorical and continuous variable analyses in this study may stem from a combination of methodological heterogeneity and sample limitations. The NLR cutoff values ​​used in the included studies varied considerably, and the methods used to determine them differed. This heterogeneity in the definition of the cutoff value directly led to high uncertainty in the pooled results of the categorical analysis. While the continuous variable analysis avoided cutoff value selection bias, its statistical power was low due to the limited number of included studies and insufficient total sample size, making it difficult to detect the true effect. The fact that the continuous results became significant after excluding the study by Li et al. (2023) in the sensitivity analysis further indicates that the current evidence system is abnormally sensitive to individual study data, resulting in insufficient robustness of the conclusions. Future research needs to develop unified NLR detection standards, standardize cutoff value determination methods, and expand multi-center sample sizes to effectively improve the comparability and explanatory power of the results.

NLR refers to the ratio between the neutrophil count and lymphocyte count in peripheral blood routine. When there is acute inflammation in the body, the number of neutrophils in peripheral blood increases abnormally, and once the body’s immune defense ability decreases, the lymphocyte count in peripheral blood routine decreases abnormally. Therefore, NLR serves as a direct indicator of the body’s acute inflammatory response and immune defense status (36–38). Recent advances have considerably enhanced our understanding of the prognostic relevance of the NLR. Numerous studies have demonstrated that an abnormally elevated NLR is strongly associated with disease severity, prognosis, and recovery outcomes. Studies have found that peripheral blood NLR>5 is positively correlated with postoperative mortality 5 days after hip fracture surgery (39). Some studies have found that postoperative NLR ≥ 4.7 increases the risk of death in individuals with hip fractures (40). Research has indicated a strong correlation between NLR and inflammatory diseases such as ulcerative colitis, acute appendicitis, cardiovascular disease, colorectal cancer and lung cancer (41–45). Concurrently, some studies also have indicated that NLR surpasses white blood cell and neutrophil counts in predicting adverse outcomes in individuals with cardiovascular diseases and malignant tumors (41, 42, 44).

In addition, inflammation is the most important stress response of the body in the development of osteoporosis. Regardless of the type of osteoporosis, the common feature is that it is accompanied by systemic or focal inflammatory response. The inflammatory response in the body will enhance the activity of osteoclasts, leading to increased bone resorption, breaking the original bone homeostasis in the body, and thus accelerating the development of osteoporosis (46, 47). Barbour et al. (48) found that abnormally elevated inflammatory markers increased the risk of hip fracture in elderly women. These studies collectively highlight the significant role of the body’s inflammatory response in the pathogenesis of osteoporosis. Furthermore, a large body of clinical studies have confirmed that the peripheral blood NLR ratio plays a crucial role in fracture risk. In a study involving 438 individuals, the bone density of the lumbar spine and femoral neck decreased with the increase of NLR (49). An increased neutrophil-to-lymphocyte ratio (NLR) after a hip fracture has been recognized as an indicator of postoperative mortality and cardiovascular events (39). Atlas et al. (50) observed that NLR levels were elevated in patients admitted to the intensive care unit (P = 0.007) as well as in those who succumbed (P = 0.007) within 5 days following hip fracture surgery. The significant associations observed in this meta-analysis support the role of the aforementioned inflammatory mechanisms in vertebral fractures.

When assessing the specificity of NLR as a predictive biomarker for vertebral fractures, the potential confounding effects of common complications in elderly patients with osteoporosis, such as cardiovascular disease, diabetes, and chronic kidney disease, on NLR levels must be fully considered. These complications are often accompanied by a chronic low-grade inflammatory state, which can independently cause neutropenia or lymphopenia, leading to non-specific increases in NLR values ​​and thus obscuring the true causal relationship between NLR and vertebral fracture risk. For example, patients with cardiovascular disease have higher underlying inflammation levels, and their elevated NLR may reflect vascular inflammation processes rather than simple bone metabolic abnormalities. Therefore, future research needs to use rigorous multivariate adjusted models or stratified analyses targeting specific comorbidity populations to control for these confounding factors in order to more accurately assess the specific predictive ability of NLR for vertebral fractures and avoid overestimating its predictive value.

This study has several limitations that require careful consideration. First, the sample size is relatively small and the geographical distribution of the studies is highly concentrated, with most studies involving Asian populations, which limits the generalizability of the results to a wider population. Second, there are significant differences in the selection of cutoff values ​​for NLR among the studies, and the lack of a unified standard may introduce classification bias and increase heterogeneity. Furthermore, the diagnostic criteria for vertebral fractures are inconsistent across different studies, relying on imaging examinations in some cases and combining clinical symptoms in others; this inconsistency in judgment criteria may affect the pooling and comparison of results. Statistically, most original studies did not adequately adjust for potential confounding factors, such as patient comorbidities, medication history, and lifestyle, which may interfere with the observed associations. Finally, the limited number of included studies, and the tendency for positive results to be more easily published, may introduce publication bias and potentially affect the estimation of the pooled effect size. Despite these limitations, this meta-analysis provides preliminary evidence consolidation of the association between NLR and the risk of vertebral fractures, and further validation with more high-quality, large-sample prospective studies is needed.

## 5 Conclusion

This systematic review and meta-analysis is the first to comprehensively assess the association between NLR and the risk of vertebral fracture in patients with osteoporosis. The results showed that, based on categorical variable data, a higher NLR was significantly associated with an increased risk of vertebral fracture; however, continuous variable data did not show a significant difference in NLR levels between the fracture and non-fracture groups. This inconsistency highlights the significant heterogeneity among studies and its underlying limitations, including a limited number of included studies, insufficient overall sample size, geographical limitations due to the majority of studies focusing on Asian populations, inconsistent NLR cutoff values ​​used across studies, differences in diagnostic criteria for vertebral fracture, and insufficient control over potential confounding factors. These factors suggest that the strength of the current evidence should be viewed with caution. Future research should focus on large-scale, multicenter prospective cohort studies, prioritizing the standardization of NLR measurement procedures, combining NLR with commonly used clinical indicators to assess its incremental predictive value, including broader populations to validate the generalizability of the results, and conducting long-term follow-up to clarify the long-term prognostic significance of NLR. In conclusion, the clinical application value of NLR as a potential biomarker for vertebral fractures has begun to emerge, but its exact clinical efficacy still needs to be further verified by higher-quality studies.

## Data Availability

The original contributions presented in the study are included in the article/**Supplementary Material**. Further inquiries can be directed to the corresponding author.
