# Supplementary material for: Association between neutrophil to lymphocyte ratio and the risk of vertebral fracture in patients with osteoporosis: a systematic review and meta-analysis
Source: Front Endocrinol (Lausanne). 2026 Feb 18;17:1739898. doi: 10.3389/fendo.2026.1739898 (PMC12956537; doi:10.3389/fendo.2026.1739898)

TableS1 Literature search strategy

Pubmed-9

((((("Neutrophils"[Mesh]) OR ((((Neutrophil) OR (Polymorphonuclear Leukocyte)) OR (LE Cell)) OR (Neutrophil Band Cell))) AND (("Lymphocytes"[Mesh]) OR (((Lymphocyte) OR (Lymphoid Cells)) OR (Lymphoid Cell)))) AND (Ratio)) AND (("Osteoporosis"[Mesh]) OR (Osteoporoses))) AND (("Fractures, Bone"[Mesh]) OR (((((Bone Fracture) OR (Broken Bones)) OR (Broken Bone)) OR (Fracture)) OR (Fractures)))

Embase-30

((Neutrophils or (Neutrophil or Polymorphonuclear Leukocyte or LE Cell or Neutrophil Band Cell)) and (Lymphocytes or (Lymphocyte or Lymphoid Cells or Lymphoid Cell)) and Ratio and (Osteoporosis or Osteoporoses) and (Fractures, Bone or (Bone Fracture or Broken Bones or Broken Bone or Fracture or Fractures))).af.

Cochrane-0

((Neutrophils or (Neutrophil or Polymorphonuclear Leukocyte or LE Cell or Neutrophil Band Cell)) and (Lymphocytes or (Lymphocyte or Lymphoid Cells or Lymphoid Cell)) and Ratio and (Osteoporosis or Osteoporoses) and (Fractures, Bone or (Bone Fracture or Broken Bones or Broken Bone or Fracture or Fractures))).af.

Web of science-22

(((((Neutrophils) OR ((((Neutrophil) OR (Polymorphonuclear Leukocyte)) OR (LE Cell)) OR (Neutrophil Band Cell))) AND ((Lymphocytes) OR (((Lymphocyte) OR (Lymphoid Cells)) OR (Lymphoid Cell)))) AND (Ratio)) AND ((Osteoporosis) OR (Osteoporoses))) AND ((Fractures, Bone) OR (((((Bone Fracture) OR (Broken Bones)) OR (Broken Bone)) OR (Fracture)) OR (Fractures))) (Topic)

Wanfang-10


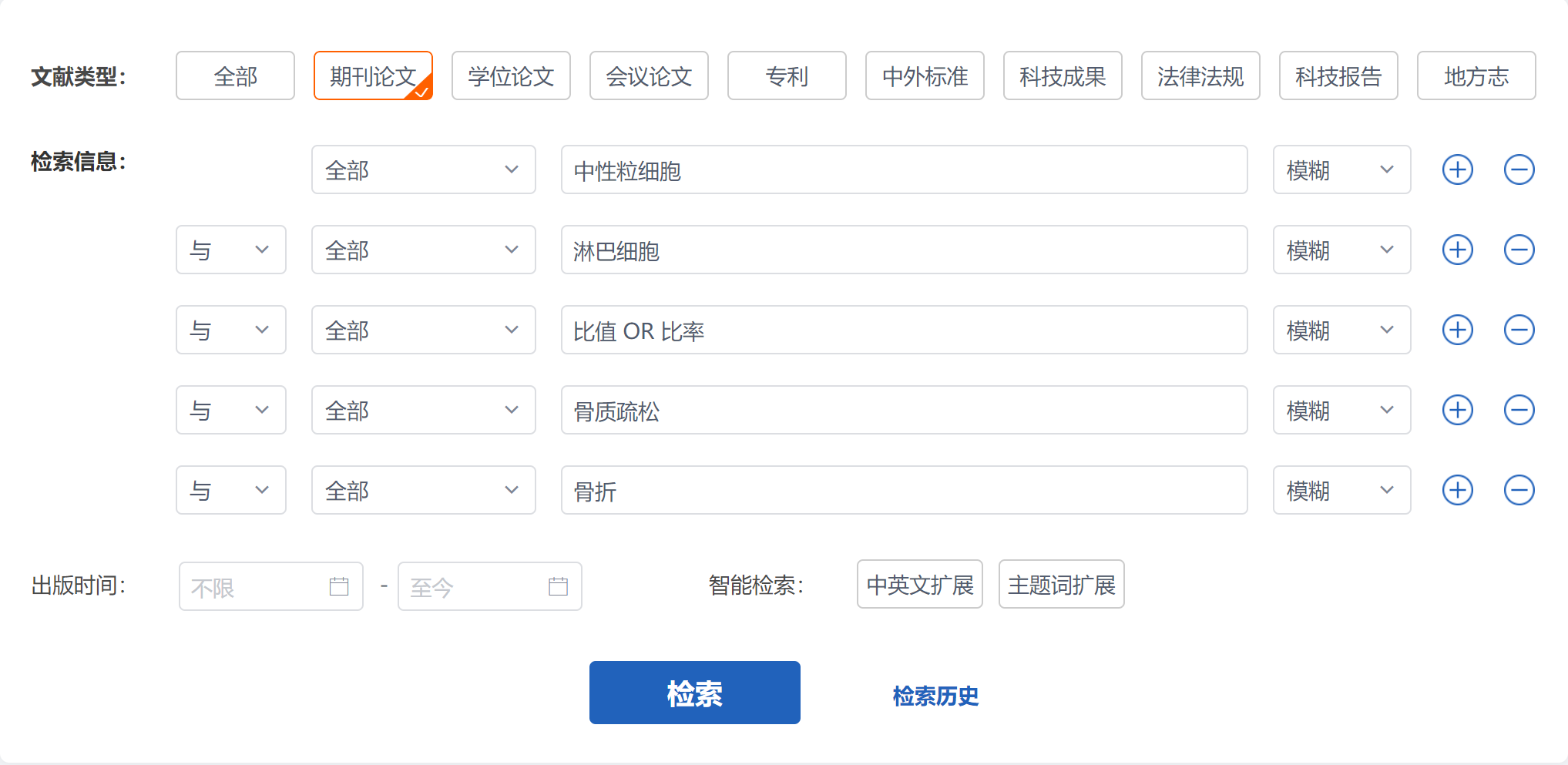


CNKI-9


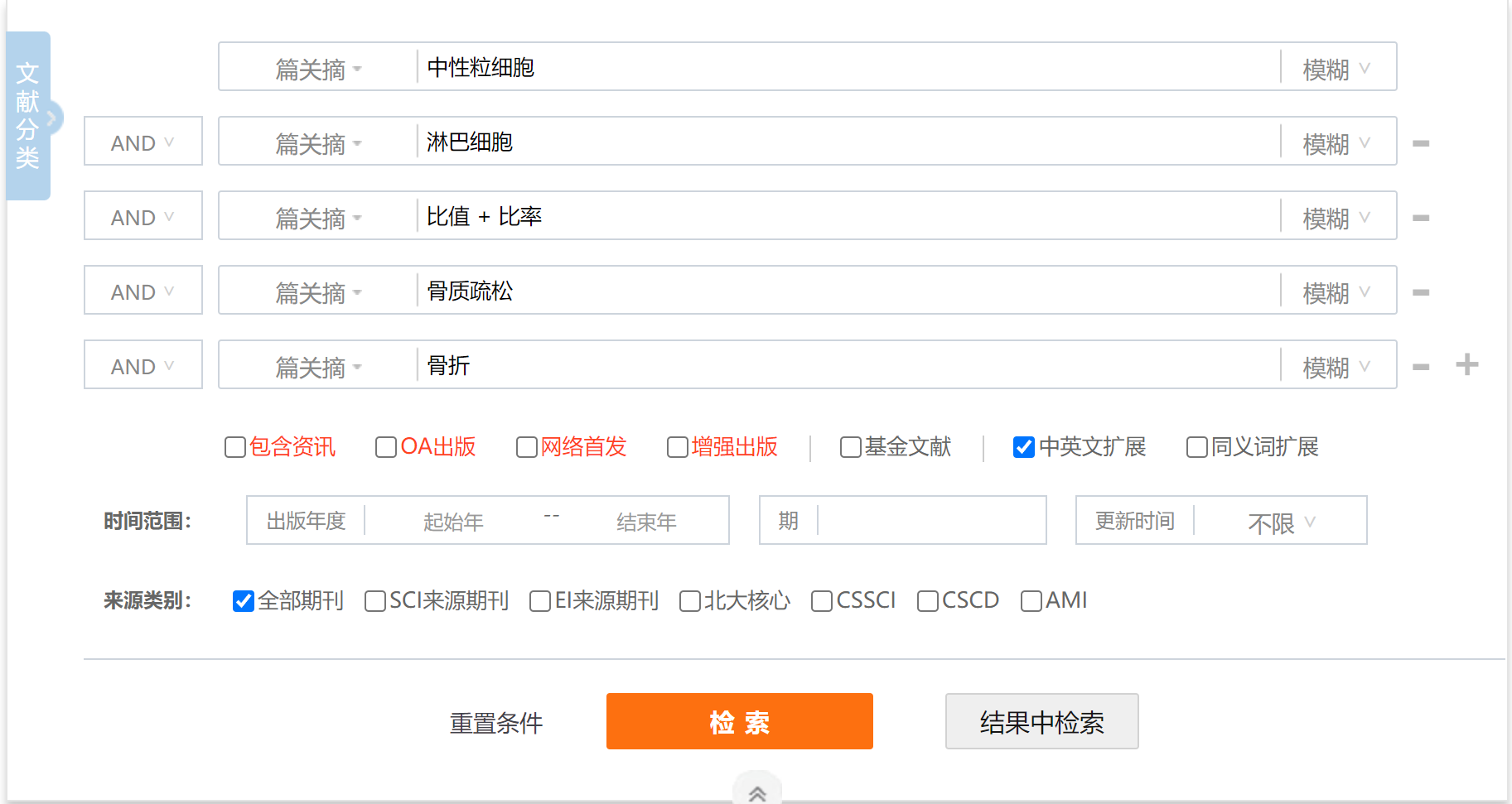

Supplement: Supplementary file 1 [file Table1.docx]
